# Perceived Teacher Support Profiles and Students’ Mathematics Engagement, Anxiety and Attitude: A Latent Profile Analysis

**DOI:** 10.3390/bs15111578

**Published:** 2025-11-18

**Authors:** Yu Zhou, Bin Jing, Hongliang Ma, Hongchao Liu

**Affiliations:** Faculty of Education, Shaanxi Normal University, Xi’an 710062, China; zhouyu@snnu.edu.cn (Y.Z.); jingbin@snnu.edu.cn (B.J.); mahl@snnu.edu.cn (H.M.)

**Keywords:** perceived teacher support, mathematics engagement, mathematics anxiety, mathematics attitude, latent profile analysis

## Abstract

The importance of perceived teacher support in mathematics learning is well-documented, yet individual student differences have often been overlooked. This study examined Chinese high school students in a highly standardized educational system characterized by a uniform curriculum, competitive rankings, and high-stakes examinations. We adopted a person-centered approach and analyzed perceptions from a sample of 1314 students, identifying three profiles: low (5.78%), medium (44.29%), and high (49.93%) perceived levels of teacher support. Results showed that neither gender nor grade predicted profile membership; however, significant variations emerged in mathematics engagement, anxiety, and attitude. Further analysis revealed significant differences across these profiles in behavioral, cognitive, and emotional engagement, as well as in classroom anxiety, learning motivation, and learning strategy. Mediation analysis demonstrated that mathematics attitude indirectly linked perceived teacher support to mathematics engagement, whereas anxiety did not mediate this relationship. These findings underscore how individual differences in perceived teacher support influence mathematics engagement, anxiety, and attitude. Stronger support fosters a more positive attitude and greater engagement, providing empirical support for differentiated instruction.

## 1. Introduction

Perceived teacher support significantly influences student learning outcomes in mathematics education (H. Lei et al., 2018; Tao et al., 2022; Z. Wang et al., 2021). Previous studies have shown that students’ perceptions of teacher support often vary by gender (Bayram Özdemir & Özdemir, 2020; H. Lei et al., 2018), while others emphasize distinctions by grade (Rautanen et al., 2022; Rice et al., 2013). Further research has indicated that effective teacher support not only alleviates students’ mathematics anxiety but also fosters a positive learning attitude and enhances engagement in mathematics (R. D. Liu et al., 2018; Ober et al., 2021; Z. Wang et al., 2021). Therefore, a thorough understanding of the mechanisms through which students perceive teacher support is essential for improving the quality of mathematics instruction.

However, most existing studies have adopted variable-centered approaches that assume relative homogeneity among students (Hickendorff et al., 2018; J. Zhou & Hawrot, 2023). In contrast, developmental context theory posits that students’ perceptions of teacher support are likely to be heterogeneous (Lerner, 2018), underscoring the need for a more nuanced, person-centered analytical approach. Therefore, it is critical to examine how different profiles of perceived teacher support relate to student characteristics (such as gender and grade) and how these perception patterns influence mathematics engagement, anxiety, and attitude.

Person-centered approaches effectively capture individual differences in students’ perceptions of teacher support (Laursen & Hoff, 2006; Shin & Chang, 2022). In this study, we employed latent profile analysis (LPA) to identify distinct profiles of perceived teacher support. This method not only reveals the diversity and complexity of students’ perceptions but also provides a foundation for understanding their potential effects (Hickendorff et al., 2018; Shin & Chang, 2022). Grounded in self-determination theory (Deci & Ryan, 2000; Ryan & Deci, 2017), we further examined how these support profiles relate to key learning variables, aiming to clarify their impact on mathematics learning processes. The findings offer person-centered insights into the role of perceived teacher support in mathematics education and can inform the development of tailored teaching strategies to improve student educational outcomes.

## 2. Literature Review

### 2.1. Perceived Teacher Support and Mathematics Learning

Perceived teacher support refers to the care and assistance that students perceive from teachers within the educational environment, encompassing three aspects: academic support, emotional support, and competence support (X. Liu et al., 2021; Ouyang & Song, 2005). In mathematics education, academic support is reflected in teachers’ attentiveness to students’ learning progress and timely help when they encounter difficulties. Emotional support includes teachers’ positive encouragement, unconditional acceptance, genuine respect, and strong trust in students. Meanwhile, competence support involves teachers encouraging students to engage in extracurricular activities and mathematics competitions (Federici & Skaalvik, 2014; X. Liu et al., 2021; Ouyang & Song, 2005).

Perceived teacher support serves as a crucial catalyst for enhancing students’ mathematics learning outcomes (Ryan & Deci, 2017; Shin & Chang, 2022). Grounded in self-determination theory, teacher support can meet students’ basic psychological needs for competence, autonomy, and relatedness, thereby fostering learning motivation and effectiveness (Deci & Ryan, 2000; Ryan & Deci, 2017). Positive teacher–student interactions that respond to students’ needs promote mathematics learning (Gan & Peng, 2024; Tao et al., 2022; Yang et al., 2021). Studies have shown that this kind of support not only has a positive impact on students’ learning motivation, cognitive processes, learning behaviors, and emotional experiences (H. Li et al., 2023; Yang et al., 2024; Y. Zhou et al., 2025), but also encourages students to participate more deeply and actively in mathematics learning by enhancing their emotional engagement and improving their learning attitude (Chen et al., 2023; H. Li et al., 2021; R. Luo et al., 2023), ultimately contributing to improved academic achievement in mathematics.

### 2.2. Gender, Grade, and Perceived Teacher Support

Regarding the relationship between students’ gender and perceived teacher support, existing studies have produced mixed conclusions (Bayram Özdemir & Özdemir, 2020; Lennon-Maslin & Quaiser-Pohl, 2024). Some studies have suggested that girls tend to report higher levels of their perceived support from teachers than boys (H. Lei et al., 2018; Wentzel et al., 2017), a difference often interpreted as girls paying more attention to interpersonal intimacy and thus being more sensitive to teachers’ emotional and behavioral support (Ewing & Taylor, 2009). However, other scholars proposed the opposite based on the vulnerability hypothesis, arguing that boys, who are more likely to face challenges in academic or behavioral adaptation, may receive greater attention and support from teachers and thus perceive stronger external assistance (Ewing & Taylor, 2009; Hamre & Pianta, 2001). Notably, some studies have found no significant gender differences in perceived teacher support (Bayram Özdemir & Özdemir, 2020; Hughes & Cao, 2018), indicating that this issue requires further clarification.

At the grade level, there are dynamic changes in students’ perceptions of teacher support. Studies have shown that the perceived intensity of teacher support varies across students at different educational stages. For example, fifth-grade students and college students report higher levels of teacher support than students in other grades (Rice et al., 2013). Other longitudinal or cross-grade studies have indicated that as students transition from lower to higher grades, perceived support from teachers tends to decline gradually, with younger students typically experiencing more supportive behaviors from teachers (Rautanen et al., 2022). This grade-level disparity may be closely related to students’ cognitive development, changes in the educational environment, and shifts in teacher–student interaction patterns.

Taken together, these inconsistent findings highlight the need for further exploration of how gender and grade level influence students’ perceptions of teacher support.

### 2.3. Perceived Teacher Support and Mathematics Engagement, Anxiety, and Attitude

#### 2.3.1. Perceived Teacher Support and Mathematics Engagement

Mathematics engagement refers to the comprehensive manifestation of students’ behavioral intensity, emotional states, and cognitive strategies during mathematics learning, encompassing three dimensions: behavioral engagement, cognitive engagement, and emotional engagement (Fredricks et al., 2004; R. D. Liu et al., 2018). Among them, behavioral engagement is reflected in students’ active listening, participation in activities, and contribution to classroom discussions. Cognitive engagement involves students’ use of deep learning strategies, such as forming connections between new and prior knowledge, exploring new problem-solving approaches, and evaluating the skills they have mastered. Emotional engagement describes students’ emotional responses (positive or negative) to learning mathematics, including attitudes toward education, sense of belonging, interest in learning, and related emotional experiences (Fredricks et al., 2004; R. D. Liu et al., 2018).

Empirical studies have shown that perceived teacher support has a positive promoting effect on students’ mathematics engagement (P. Li et al., 2025; R. D. Liu et al., 2018; Y. Zhou et al., 2025). In particular, teacher support helps build positive teacher–student relationships, optimizes the teaching process, and stimulates students’ learning motivation. In this supportive environment, students tend to exhibit higher enthusiasm and confidence in learning, enabling them to focus more on mathematical tasks and actively participate in classroom interactions (Gan & Peng, 2024; H. Li et al., 2023). Furthermore, teacher support can significantly enhance students’ engagement across behavioral, cognitive, and emotional dimensions (Tao et al., 2022; Yang et al., 2021, 2024). From the perspective of self-determination theory, teacher support satisfies students’ basic psychological needs (e.g., competence, autonomy, and relatedness), thereby promoting more active engagement in learning activities and contributing to higher levels of academic achievement and personal development (H. Li et al., 2023; Rautanen et al., 2022; Ryan & Deci, 2017).

#### 2.3.2. Mathematics Anxiety as a Mediator

Mathematics anxiety refers to the tension, distress, and fear that individuals experience when engaging in mathematics-related tasks. Depending on the context, mathematics anxiety can be categorized into three types: problem-solving anxiety, test anxiety, and classroom anxiety, which, respectively, correspond to the anxiety students feel when solving mathematics problems, taking mathematics exams, and participating in mathematics classroom activities (Beilock & Maloney, 2015; W. Lei et al., 2021; Zhang, 2021).

Studies have shown that during the process of learning mathematics, perceived teacher support effectively alleviates students’ anxiety in problem-solving, test-taking, and classroom performance (Chen et al., 2023; R. Luo et al., 2023; Z. Wang et al., 2021). Further empirical analyses indicated that higher levels of teacher support are associated with greater academic enjoyment that students experience, while levels of academic helplessness and mathematics anxiety are reduced (H. Li et al., 2021; Z. Wang et al., 2021). Furthermore, research has confirmed that mathematics anxiety is a key factor affecting students’ mathematics engagement (H. Li et al., 2023; Quintero et al., 2022; Y. Zhou et al., 2025). Compared with their highly anxious peers, students with lower mathematics anxiety are more willing to actively participate in mathematics learning and show less avoidance or procrastination tendencies when facing difficult tasks (Aldrup et al., 2020; Demedts et al., 2022). These findings suggest that mathematics anxiety may serve as a mediating psychological mechanism, constituting a key “bridge” in the relationship between perceived teacher support and student engagement.

#### 2.3.3. Mathematics Attitude as a Mediator

Mathematics attitude refers to a comprehensive psychological state of thinking, feeling, perception, and behavioral tendencies exhibited by an individual in the process of learning and applying mathematics (He & Li, 2013). It typically comprises three dimensions: learning motivation, learning belief, and learning strategy. Among these, learning motivation mainly arises from an interest in mathematics; learning belief involves students’ perceptions of and confidence in the essence of mathematical knowledge and its learning processes; and learning strategy reflects the cognitive styles and methods students adopt in mathematics learning (He & Li, 2013; Lin & Huang, 2016; Lv et al., 2025).

Empirical research has demonstrated that teacher support plays a crucial role in shaping students’ attitudes toward mathematics. Students who perceive stronger support from their teachers tend to display greater self-confidence and a more positive learning attitude (Lee & Simpkins, 2021; Yang et al., 2021). This support not only enhances students’ learning motivation but also encourages them to adopt more effective learning strategies, thereby further optimizing their mathematics attitude (Ober et al., 2021; Vidić & Đuranović, 2020). Additionally, research has indicated a close connection between mathematics attitude and students’ mathematics engagement. Students with a positive attitude toward mathematics typically exhibit stronger intrinsic motivation and learning confidence, actively apply efficient learning strategies, and excel in autonomous learning (Lin & Huang, 2016; R. Luo et al., 2023). This positive attitude further motivates students to engage more deeply in mathematics activities and the exploration process (Z. Luo & Luo, 2022; Vidić & Đuranović, 2020). Based on the above analysis, it can be concluded that mathematics attitude may mediate the relationship between perceived teacher support and mathematics engagement.

### 2.4. The Need for a Person-Centered Approach to Profile Perceived Teacher Support

Previous studies have largely adopted a variable-centered approach to exploring the relationship between perceived teacher support and factors related to mathematics learning. This approach assumes homogeneous effects of perceived teacher support on mathematics learning across students (Hickendorff et al., 2018; Laursen & Hoff, 2006). However, while this perspective simplifies reality, it also treats teacher support as a homogeneous variable, failing to fully reflect the individual differences and uniqueness that are widespread among student groups. Developmental-context theory emphasizes that person–environment interactions are dynamic and diverse. In educational settings, students actively interpret and respond to teacher support based on their own cognition, emotions, and needs, forming unique developmental trajectories rather than passively receiving support (Lerner, 2018). This theory highlights individual heterogeneity, making students’ perceptions of teacher support highly individualized. Different students may experience completely different perceptions and responses to the same form and degree of support, which in turn leads to divergent developmental outcomes (Howard & Hoffman, 2018). Therefore, the traditional variable-centered research hypothesis may overlook the substantial heterogeneity present in students’ actual perceptions of teacher support (Bergman et al., 2015; Zhu et al., 2024), and research conclusions may not accurately reflect educational reality or provide targeted guidance for teaching practice.

The person-centered approach offers a new perspective for understanding the heterogeneity of students’ perceptions of teacher support. This method can identify student profiles with similar perceptual patterns and examine differences in mathematics learning outcomes across different profiles (Laursen & Hoff, 2006). LPA, an effective tool within this approach, reveals potential relationships between key variables by identifying individuals with similar characteristics, highlighting individual differences and developmental stages, and tracking group heterogeneity (Hickendorff et al., 2018). Although person-centered approaches provide significant advantages in revealing individual differences, this does not mean that variable-centered approaches should be replaced (Wormington & Linnenbrink-Garcia, 2017). The two are complementary: variable-centered methods excel at accurately quantifying predictor variables and elucidating relationships through statistical analysis, while person-centered approaches more comprehensively assess the complexity and interaction of relationships among predictors, grounded in the uniqueness of individuals or groups (Lanza & Cooper, 2016; Xu, 2024). Thus, person-centered analyses can provide a valuable complement to variable-centered studies.

To summarize, research on perceived teacher support that accounts for student heterogeneity is still limited. It remains unclear what the perceived teacher support profiles are and whether students’ characteristics (e.g., gender and grade) influence perceived teacher support latent profiles. In particular, the questions of how different perceived teacher support profiles relate to and what mechanisms underlie mathematics learning outcomes remain unresolved. Consequently, it is necessary to adopt a person-centered approach combined with LPA to conduct an in-depth examination of students’ perceptions of teacher support and to explore how these profiles relate to mathematics-learning–related factors.

## 3. The Present Study

This research examined perceived teacher support from a person-centered perspective, utilizing the LPA approach to identify latent profiles of perceived teacher support. Additionally, the study investigated the relationships between these profiles and various factors, including gender, grade, engagement, anxiety, and attitudes toward mathematics. Furthermore, we explored the underlying relationships and mechanisms between different perceived teacher support profiles and engagement, anxiety, and attitude toward mathematics. Given the gaps identified in existing research, this study addresses four specific questions (RQs):

RQ1. What are the characteristics of the latent perceived teacher support profiles?

RQ2. Do gender and grade predict different perceived teacher support profiles?

RQ3. Are differences in engagement, anxiety, and attitudes toward mathematics among students related to different perceived teacher support profiles?

RQ4. What are the relationships and mechanisms between different perceived teacher support profiles and engagement, anxiety, and attitudes toward mathematics?

## 4. Method

### 4.1. Participants

This study surveyed students in grades 10 to 12 from three public high schools in China. Within the Chinese education system, mathematics education maintains coherence and integrity in the knowledge system by using unified teaching materials and instructional plans. Teacher training programs aim to provide all students with high-quality instruction, while consistent teaching standards help students follow similar and efficient learning paths under a unified curriculum. Classes are organized based on academic achievement, which acknowledges differences between classes while ensuring that students within the same class perform at similar levels. Class size is maintained at 55 students to optimize teaching management. Additionally, a unified examination and ranking system offers a fair competitive environment for students and supports teachers and education departments in assessing learning progress.

This study surveyed 1350 online questionnaires, obtaining 1314 valid questionnaires, with an effective rate of 97.33%. Among them, 563 were completed by boys (42.85%) and 751 were girls (57.15%), with an average age of 17.50 ± 1.18 years. There were 736 students in 10th grade (56.01%), 520 in 11th grade (39.57%), and 58 in 12th grade (4.42%).

### 4.2. Data Collection

In the data collection stage, we strictly adhered to the principle of informed consent and obtained consent from school administrators, surveyed students, and their guardians. Students participated voluntarily and could withdraw at any time. In addition, we used anonymous questionnaires to protect privacy. With the assistance of the school, students completed an online questionnaire through WJX (www.wjx.cn), covering perceived teacher support, mathematics engagement, anxiety, and attitude scales, which took approximately 30–45 min. Before completing the questionnaire, the researcher informed the students that the purpose of the survey was for academic research only, namely to understand their overall experience and feelings in the process of learning mathematics, and emphasized objective evaluation and honest feedback. The teacher would not know the results provided by the students. After the questionnaire survey was completed, the data were strictly cleaned, and unqualified responses were eliminated according to predefined criteria to ensure the objectivity and validity of the results. The criteria included the following: (1) more than half of the questions had the same or missing answers; (2) the response time for each question was less than two seconds (DeSimone et al., 2015; J. L. Huang et al., 2012).

### 4.3. Measures

The reliability of each questionnaire was analyzed using Cronbach’s α coefficients, and its structural validity was evaluated by confirmatory factor analysis (CFA; see Table 1). These results were final after removing underloaded factors. For reliability, the recommended *α* is greater than 0.70 (Cronbach, 1951). For CFA, the fit metrics that must be reported in general studies include the ratio of chi-square to degrees of freedom (*χ*^2^/*df*), the comparative fit index (*CFI*), the Tucker–Lewis index (*TLI*), the root-mean-square error of approximation (*RMSEA*), and the standardized root mean residual (*SRMR*). The recommended fitting indexes in the literature are *CFI* ≥ 0.90, *TLI* ≥ 0.90, *RMSEA* ≤ 0.06, and *SRMR* ≤ 0.10 (Meyers et al., 2016). Due to the large sample size, the value of *χ*^2^/*df* is too high, so this value was not used as a criterion for validity in this study.

#### 4.3.1. Perceived Teacher Support

The perceived teacher support scale was adopted, which was translated and revised by Ouyang and Song (2005) based on the original English scale of Babad (1990). This scale has been validated by X. Liu et al. (2021) in the Chinese educational context and can effectively and accurately assess Chinese students’ perceptions of teacher support. This scale has 19 items and contains three dimensions: academic support, emotional support, and competence support (e.g., “The teacher would use encouraging eyes to signal me to get up and answer questions”, “The teacher’s attitude toward me has always been very gentle”, and “The teacher often puts me in charge of things in the class”, respectively). Each item is evaluated using a 5-point Likert scale ranging from 1 (completely disagree) to 5 (completely agree), with higher scores on each dimension indicating a higher level of perceived teacher support. Through CFA, four items with factor loadings less than 0.60 were deleted.

#### 4.3.2. Mathematics Engagement

The mathematics engagement scale was adopted by R. D. Liu et al. (2018), which was translated and revised based on the original English scale by M. T. Wang et al. (2016). This scale has been validated in the Chinese educational environment and can effectively measure students’ mathematics engagement levels. It has 16 items in total, including three dimensions: behavioral engagement, cognitive engagement, and emotional engagement (e.g., “I actively engage in class discussions during mathematics class”, “When I study mathematics, I think about how the knowledge is used in real life”, and “I enjoy learning new knowledge in mathematics class”, respectively). Each item is evaluated using a 5-point Likert scale ranging from 1 (completely disagree) to 5 (completely agree). The higher the score is, the greater the student’s engagement in mathematics learning.

#### 4.3.3. Mathematics Anxiety

The mathematics anxiety scale translated and revised by Zhang (2021), based on the original English scale developed by Richardson and Suinn (1972), was selected. This scale has been validated in the Chinese educational setting and can measure students’ mathematics anxiety well. It has 17 items in total, encompassing three dimensions: problem-solving anxiety, test anxiety, and classroom anxiety (e.g., “I have trouble concentrating when solving problems”, “I’m always worried about failing a mathematics test”, and “I often feel nervous and uncomfortable in mathematics class”, respectively). Each item is evaluated using a 5-point Likert scale ranging from 1 (not at all anxious) to 5 (very anxious). The higher the score, the more serious the student’s mathematics anxiety.

#### 4.3.4. Mathematics Attitude

The mathematics attitude scale developed by He and Li (2013) was used, which has been validated in the Chinese educational setting and can measure students’ attitudes toward mathematics well. It has a total of 27 items, encompassing three dimensions: learning motivation, learning belief, and learning strategy (e.g., “I’m learning mathematics because it’s fun”, “I’m sure I can do harder mathematics homework”, and “At different learning stages, I will set corresponding mathematics learning goals for myself”, respectively). Each item is evaluated using a 5-point Likert scale ranging from 1 (very inconsistent) to 5 (very consistent). The higher the score, the more positive the attitude toward mathematics learning. Through CFA, seven items with factor loadings less than 0.60 were deleted.

### 4.4. Data Analysis

This study utilized SPSS 26.0 and Mplus 8.3 for data management and analysis. To answer RQ1, we used Mplus 8.3 to conduct LPA and estimated the best model of different combination modes based on the following three criteria: (1) Lower AIC, BIC, and aBIC values indicate better model fit; (2) for LRT and BLRT, the *p*-value is significant, indicating that the k-type fitted model performs better than the k − 1 type model. Nylund (2007) noted that BLRT performs better than the LRT; (3) the closer the entropy score is to 1, the greater the probability of accurate classification of an individual. Simplicity and interpretability must also be considered in model selection. This study aimed to explore the profile of student-perceived teacher support based on LPA and tested 2 to 4 types of models to determine the best-fitting model. To answer RQ2, based on the LPA results, we used SPSS 26.0 to perform multiple logistic regression analyses and examine the effects of gender and grade on latent profiles of perceived teacher support. To answer RQ3, we used SPSS 26.0 to conduct a series of one-way ANOVAs to explore differences in mathematics engagement, anxiety, and attitude across profile groups. To answer RQ4, we further used the PROCESS macro in SPSS to explore the mediating roles of mathematics anxiety and mathematics attitude between different perceived teacher support profiles and mathematics engagement.

## 5. Results

### 5.1. Common Method Bias

Since all variables in this study were assessed using self-report questionnaires, common method bias may be a concern. To examine this potential issue, we applied the Harman single-factor test. An exploratory factor analysis (without rotation) was performed on all measurement items. The analysis revealed that the first common factor explained 29.32% of the total variance, which is below the commonly recommended threshold of 40% (H. Zhou & Long, 2004). Thus, no significant common method bias was detected.

### 5.2. RQ1: LPA Results of Perceived Teacher Support

We employed LPA to classify perceived teacher support in the sample. Model parameters are presented in Table 2. We compared fit indices for models ranging from 1 to 4 profiles: as the number of profiles increased, AIC, BIC, and aBIC all decreased. However, the decline in these indices diminished when moving from the 3- to the 4-profile model. Entropy was highest for the 2-profile model. Furthermore, the LMR test for the 4-profile model was not significant, suggesting that it did not yield a better fit than the 3-profile model. Based on goodness of fit, model simplicity, and interpretability, the 3-profile model was selected as optimal.

**Figure 1:**
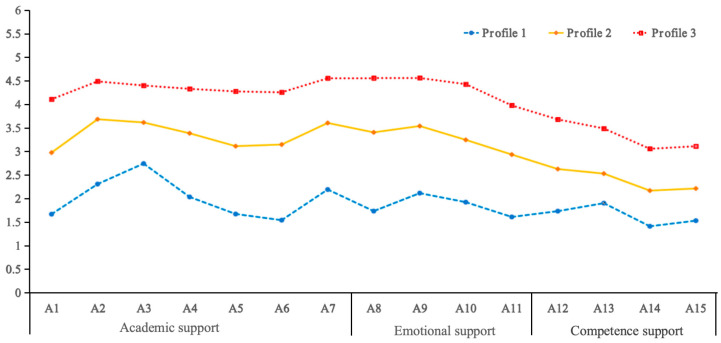
Results of LPA on perceived teacher support across academic, emotional, and competence dimensions.

Figure 1 displays the three latent profiles of perceived teacher support (Profile 1, Profile 2, and Profile 3). The LPA revealed clear differences in students’ perceptions of mathematics teacher support. Across all 15 items—categorized as academic (A1–A7), emotional (A8–A11), and competence support (A12–A15)—the three profiles showed distinct and consistent patterns. Profile 3 had the highest perceived support ratings, while Profile 1 scored the lowest. Profile 2 consistently fell between the other two profiles across all items, maintaining an intermediate position in all support dimensions. In terms of distribution, 5.78% of students were classified into Profile 1, 44.29% into Profile 2, and 49.92% into Profile 3, indicating that most students perceived medium to high levels of teacher support.

We performed a series of one-way ANOVAs with the three latent profiles as the independent variable and the three dimensions of perceived teacher support as dependent variables. As presented in Table 3, latent profile membership had a significant main effect on all three support dimensions. According to post hoc (*LSD*) tests, students in Profile 1 (low) scored significantly lower on all dimensions than those in Profiles 2 and 3, suggesting low perceived teacher support. Profile 2 students showed intermediate scores, reflecting a medium level of perceived support. Profile 3 students scored significantly higher than the other two profiles, indicating high perceived teacher support.

### 5.3. RQ2: Gender, Grade, and Perceived Teacher Support Profiles

We conducted a multiple logistic regression to further examine the characteristics of students’ perceived teacher support. The latent profiles served as the dependent variable, with gender (reference: girls) and grade (reference: 10th grade) as independent variables. Using the low-support profile as the reference group, the analysis produced odds ratios (see Table 4). Results indicated that neither gender nor grade significantly predicted profile membership, suggesting no notable demographic differences among students in the low-, medium-, and high-support profiles.

### 5.4. RQ3: The Perceived Teacher Support Profiles and Mathematics Engagement, Attitude, and Anxiety

Continue to adopt ANOVA to examine differences in mathematics engagement, anxiety, and attitude across the three perceived teacher support profiles. As shown in Table 5, students with different profiles exhibited significant differences in all three outcome variables.

Students in Profile 1 (low support) had significantly lower mathematics engagement and attitude scores than those in Profiles 2 and 3, a pattern that held across all sub-dimensions: behavioral, cognitive, and emotional engagement, as well as learning motivation and strategy. Profile 2 students (medium support) showed intermediate level across engagement, anxiety, attitude, and their subcomponents. Profile 3 students (high support) demonstrated significantly higher engagement and attitude scores, along with significantly lower mathematics and classroom anxiety, compared to the other profiles.

### 5.5. RQ4: Mechanisms Between the Perceived Teacher Support Profiles and Mathematics Engagement, Anxiety, and Attitude

We conducted a mediation analysis using Hayes’s (2017) PROCESS macro (Model 4) in SPSS to examine the potential mediating roles of mathematics anxiety and attitude in the relationship between perceived teacher support profiles and mathematics engagement. The analysis used a bootstrapping method with 5000 resamples to test indirect effects. The independent variable was the perceived teacher support profile, dummy-coded with the low-support profile as the reference (D1: medium vs. low; D2: high vs. low). Mathematics anxiety and attitude were included as parallel mediators, and mathematics engagement was the dependent variable, controlling for gender and grade.

**Figure 2:**
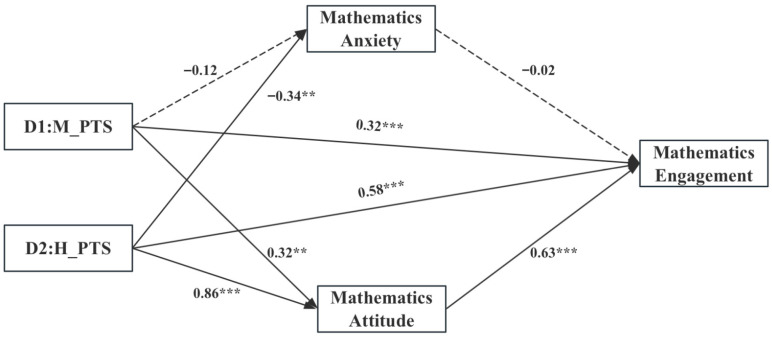
Mediation model. D1 means medium support profile compared with low support profile, D2 means high support profile compared with low support profile. N = 1314. ** *p* < 0.01, and *** *p* < 0.001. Gender and grade served as covariates. All variables in the model were standardized.

As illustrated in Figure 2, when using the low-support profile as the reference, medium support was not significantly associated with mathematics anxiety (*β* = −0.12, *p* > 0.05) but was positively associated with mathematics attitude (*β* = 0.32, *p* < 0.01) and engagement (*β* = 0.32, *p* < 0.001). High support was negatively associated with significantly lower mathematics anxiety (*β* = −0.34, *p* < 0.05) and positively higher mathematics attitude (*β* = 0.86, *p* < 0.001) and engagement (*β* = 0.58, *p* < 0.001). Mathematics anxiety was not significantly associated with engagement (*β* = −0.02, *p* > 0.05), whereas mathematics attitude was positively associated with engagement (*β* = 0.63, *p* < 0.001).

Table 6 presents the mediation results. Bootstrapped bias-corrected confidence intervals were used to test the indirect effects. The 95% CIs for the indirect pathways from perceived teacher support (medium and high, versus low) to mathematics engagement via mathematics anxiety were [−0.01, 0.02] and [−0.01, 0.04], indicating no significant mediation through anxiety. In contrast, the 95% CIs for the pathways via mathematics attitude were [0.06, 0.32] and [0.38, 0.65], supporting a significant indirect effect. Mathematics attitude thus mediated the relationship between perceived teacher support profiles and mathematics engagement, with indirect effects of 0.19 and 0.51 for the medium- and high-support profiles, respectively. These findings suggest that higher levels of perceived teacher support are associated with a more positive mathematics attitude, which in turn relates to greater mathematics engagement.

## 6. Discussion

Prior research on perceived teacher support has predominantly employed a variable-centered approach, overlooking potential distinct profiles of teacher support and their differential associations with other variables. To address this gap, the present study adopted a person-centered approach, using LPA to identify perceived teacher support profiles among high school students. We further examined how gender and grade predict profile membership and explored the relationships between these profiles and mathematics learning factors, as well as the underlying mechanisms linking profile membership to mathematics learning outcomes.

### 6.1. Three Latent Profiles of Perceived Teacher Support

This study identified three latent profiles of perceived teacher support using LPA: low (5.78%), medium (44.29%), and high (49.92%). The distribution indicates that nearly half of the students perceived a relatively high level of teacher support, while only a small proportion reported low support, suggesting an overall positive perception of teacher support among the sampled high school students. Across profiles, all three dimensions of teacher support—academic, emotional, and competence support—differed significantly, which aligns with previous research on the multidimensional structure of teacher support (X. Liu et al., 2021). Moreover, the distinct profile patterns indicate that students’ perceptions do not simply reflect isolated dimensions, but rather an integrated pattern of change across multiple support types.

From a theoretical perspective, these findings support the viewpoint of developmental context theory, which emphasizes heterogeneity in individuals’ environmental perceptions (Lerner, 2018). By adopting a person-centered approach, we identified distinct student subgroups with unique perception patterns, offering a refined framework for understanding how teacher support variably influences development. The effective application of LPA further demonstrates its utility in capturing variable interactions and individual-level manifestation patterns (Shin & Chang, 2022; J. Zhou & Hawrot, 2023).

### 6.2. Perceived Teacher Support Profiles Are Not Affected by Gender and Grade

The results showed no significant differences in perceived teacher support profiles by gender or grade level. Neither variable predicted profile membership, contrasting with some previous studies that reported demographic differences in teacher support perceptions (Bayram Özdemir & Özdemir, 2020; Hughes & Cao, 2018). This discrepancy may stem from China’s standardized education system, which emphasizes uniformity and equity, particularly under the influence of high-stakes exams such as the college entrance examination (Moon et al., 2022). In this context, teachers may prioritize collective academic achievement over individualized approaches, potentially leading to more consistent perceptions of support across gender and grade (Hughes & Cao, 2018).

Similarly, the absence of grade-level differences contrasts with certain prior findings. This may be due to the continuity emphasized in modern education systems, where stable instructional environments, objectives, and methods are maintained across grades (Moon et al., 2022). Such consistency may help students perceive similar levels of teacher support throughout high school. Furthermore, ongoing teacher professional development ensures that mathematics instructors at different grade levels provide comparable support (Thurm & Barzel, 2020). Additionally, as students advance, they often develop more stable learning attitudes and better emotional regulation, which may further homogenize their perceptions of teacher support (R. Luo et al., 2023).

### 6.3. Linking Perceived Teacher Support Profiles to Mathematics Engagement, Anxiety, and Attitude

The results revealed significant differences in mathematics engagement across the perceived teacher support profiles, with engagement increasing progressively from the low- to the high-support profile. Students in the high-support profile demonstrated significantly greater behavioral, cognitive, and emotional engagement. This finding aligns with self-determination theory, which posits that supportive teacher-student relationships fulfill psychological needs and foster engagement (Deci & Ryan, 2000; Ryan & Deci, 2017). By providing clear guidance, timely feedback, and emotional support, teachers can stimulate interest and satisfy students’ needs for autonomy and competence, thereby encouraging deeper involvement (H. Li et al., 2023; Yang et al., 2024). These results are consistent with prior variable-centered studies reporting a positive link between teacher support and engagement (Gan & Peng, 2024; Tao et al., 2022). Our person-centered approach extends this work by demonstrating that distinct support profiles are meaningfully associated with different levels of mathematics engagement, underscoring the practical value of enhancing teacher support to promote student engagement.

Significant differences in mathematics anxiety were also observed across the support profiles. Students with high perceived support reported the lowest overall anxiety, suggesting that teacher support may boost students’ confidence and reduce anxiety (R. D. Liu et al., 2018; Z. Wang et al., 2021). Further analysis indicated that profile membership significantly influenced classroom anxiety, with the high-support group showing the lowest levels (Chen et al., 2023). However, no significant differences were observed in problem-solving or test anxiety, possibly due to the inherently challenging nature of high school mathematics, which can evoke substantial pressure even among well-supported students (Kikas et al., 2020; W. Lei et al., 2021). Thus, while improving teacher support may alleviate classroom anxiety, additional strategies are needed to address anxiety related to tests and problem-solving.

Finally, students’ mathematics attitudes differed significantly across the support profiles. These differences were primarily driven by learning motivation and strategy: students with higher perceived support displayed stronger motivation and more adaptive, self-regulated learning strategies (Vidić & Đuranović, 2020; Ober et al., 2021; Yang et al., 2021). In contrast, no significant differences were found in learning belief, which may be relatively stable and less susceptible to external influences such as teacher support (Demedts et al., 2022). These findings highlight the role of teacher support in fostering a positive learning attitude, while also acknowledging the stability of core beliefs about mathematics.

### 6.4. Mathematics Attitude Is a Mediator, but Anxiety Is Not

The study revealed a strong association between perceived teacher support profiles and students’ mathematics attitudes. Both medium and high levels of support correlated with a more positive attitude, with students reporting stronger support showing greater confidence, interest, and sustained enthusiasm for mathematics (Lee & Simpkins, 2021; Ober et al., 2021). Moreover, mathematics attitude served as a significant mediator between teacher support and mathematics engagement, implying that support enhances attitudes towards learning mathematics, which in turn promotes active engagement in learning activities (R. Luo et al., 2023; Vidić & Đuranović, 2020). These findings highlight attitude as a key mechanism linking teacher support to student engagement.

Regarding mathematics anxiety, the results revealed a distinct pattern: medium support showed no significant association, while high support correlated with lower anxiety, suggesting that only substantial support mitigates anxiety effectively (H. Li et al., 2021; Z. Wang et al., 2021). However, anxiety did not mediate the support-engagement relationship, nor was it directly associated with engagement, contrasting with previous research (H. Li et al., 2023; Quintero et al., 2022). This discrepancy may stem from cultural and educational factors. In collectivist East Asian contexts, achievement emotion theory posits that students may reframe anxiety as a collective growth opportunity, potentially blunting its negative effects (Markus & Kitayama, 2014; Pekrun, 2024). Additionally, high-stakes testing systems like China’s Gaokao may normalize anxiety, integrating it into learning and weakening its typical impact on engagement (Y. Huang, 2023), which could explain the non-significant results.

## 7. Limitations and Future Directions

This study offers an innovative, person-centered perspective on mathematics education by examining students’ perceived teacher support. However, several limitations should be acknowledged, pointing to directions for future research.

First, although the study revealed associations between perceived teacher support and mathematics learning factors, its cross-sectional design precludes causal inferences and cannot track potential changes in profile membership over time. Future research should adopt longitudinal designs to examine the stability of these profiles and possible transitions between them. Second, although the person-centered approach identified distinct student profiles, it may not capture qualitative differences among students within the same profile. Future studies could combine person-centered and variable-centered methods to better address this complexity. Third, the reliance on self-report measures may introduce biases such as recall inaccuracy. Future research could improve data quality by incorporating multiple assessment time points and situational prompts into questionnaires, as well as using mixed methods (e.g., interviews, observations) to gain a more comprehensive understanding. Finally, while these findings may be relevant in educational contexts similar to China’s, further validation in diverse cultural settings is needed to enhance generalizability.

## 8. Conclusions and Implications

This study adopted a person-centered approach to explore students’ perceptions of mathematics teacher support, offering a new methodological approach that addresses the limitations of prior variable-centered research, which often overlooked student heterogeneity. Using LPA, we identified three distinct profiles of perceived teacher support: low, medium, and high. Although profile membership did not vary by gender or grade, it showed meaningful associations with mathematics engagement, anxiety, attitude, learning motivation, and strategy. Moreover, the mathematics attitude mediated the relationship between medium and high support profiles and engagement. These findings enrich the existing literature by providing a more nuanced understanding of how teacher support functions across student subgroups.

This study offers several practical implications. First, the identified profiles highlight the importance of providing fair and individualized support to foster positive teacher–student relationships and constructive learning experiences (Moon et al., 2022). Second, given that higher perceived support is associated with improved engagement, a more positive attitude, and reduced anxiety, teachers should intentionally incorporate diverse support strategies into their practice (Gan & Peng, 2024; Z. Wang et al., 2021). Notably, the mediating role of mathematics attitude suggests that enhancing support can help shape positive attitudes, stimulate interest, and boost engagement (R. Luo et al., 2023; Z. Luo & Luo, 2022). In summary, mathematics teachers should tailor support to students’ abilities, personalities, and learning styles, ensuring all students feel valued. Establishing an inclusive classroom atmosphere can help prevent inequitable support, thereby reducing anxiety, encouraging a positive attitude, and promoting active engagement in learning mathematics.

## Figures and Tables

**Table 1:** Reliability and validity of measurement tools.

Scale	Dimension	Cronbach’s α	χ^2^/*df*	CFI	TLI	RMSEA	SRMR
Perceived teacher support		0.93	6.57	0.94	0.92	0.07	0.06
	Academic support	0.90					
	Behavior support	0.88					
	Competence support	0.87					
Mathematics engagement		0.96	7.11	0.94	0.92	0.07	0.05
	Behavioral engagement	0.92					
	Cognitive engagement	0.94					
	Emotional engagement	0.95					
Mathematics anxiety		0.95	8.09	0.92	0.91	0.07	0.07
	Problem-solving anxiety	0.92					
	Test anxiety	0.92					
	Classroom anxiety	0.92					
Mathematics attitude		0.80	3.93	0.96	0.95	0.05	0.07
	Learning motivation	0.92					
	Learning beliefs	0.86					
	Learning strategies	0.92					

Note: *df* = degrees of freedom; CFI = comparative fit index; TLI = Tucker–Lewis index; RMSEA = root mean square error of approximation; SRMR = standardized root mean square residual.

**Table 2:** Fit indices for the four models using the LPA approach (N = 1314).

Profile Model	AIC	BIC	aBIC	Entropy	LMR	BLRT	Group Size for Each Profile			
							1	2	3	4
1-profile model	62,210.94	62,366.37	62,271.07	-	-	-	1314			
2-profile model	55,806.19	56,044.51	55,898.39	0.933	0.000	0.000	545	769		
3-profile model	53,671.35	53,992.56	53,795.62	0.931	0.043	0.000	76	582	656	
4-profile model	52,029.17	52,433.27	52,185.50	0.932	0.161	0.000	440	41	571	262

**Table 3:** Descriptive statistics, ANOVA, and Post hoc (*LSD*) tests for the three latent profiles of perceived teacher support on three-dimensional scores.

Profile	Academic Support	Emotional Support	Competence Support
Profile 1 (76)	1.67 ± 0.80	1.45 ± 0.80	1.25 ± 0.89
Profile 2 (582)	3.17 ± 0.44	3.09 ± 0.53	2.06 ± 0.89
Profile 3 (656)	4.30 ± 0.47	4.34 ± 0.50	3.15 ± 1.08
*F*(2,1314)	1518.00 ***	1502.96 ***	260.19 ***
Post hoc (*LSD*)	1 < 2 < 3	1 < 2 < 3	1 < 2 < 3
Effect size	0.698	0.696	0.284

Note: *** *p* < 0.001.

**Table 4:** Multiple logistic regression of the gender and grade variables on latent profiles of perceived teacher support.

		Profile 2			Profile 3		
		OR	95%CI		OR	95%CI	
Gender	Male	0.99	0.60	1.61	1.27	0.78	2.06
	Female	1			1		
Grade	10	1.06	0.24	4.80	0.52	0.12	2.24
	11	0.74	0.17	3.35	0.32	0.08	1.39
	12	1			1		

**Table 5:** ANOVA and post hoc (*LSD*) tests for the three latent profiles of perceived teacher support on mathematics engagement, anxiety, and attitude.

	Profile M (*SD*)			*F*(2,1314)	Effect Size	Post Hoc (*LSD*)
	Profile 1	Profile 2	Profile 3			
ME	2.95 ± 0.88	3.33 ± 0.64	3.77 ± 0.69	92.80 ***	0.124	1 < 2 < 3
ME_BE	3.19 ± 0.94	3.49 ± 0.68	3.86 ± 0.70	58.99 ***	0.083	1 < 2 < 3
ME_CE	2.80 ± 1.02	3.15 ± 0.74	3.62 ± 0.81	73.44 ***	0.101	1 < 2 < 3
ME_EE	2.84 ± 0.97	3.35 ± 0.76	3.82 ± 0.81	87.83 ***	0.118	1 < 2 < 3
MA	3.20 ± 0.10	3.10 ± 0.04	2.95 ± 0.03	11.83 ***	0.009	3 < 2 < 1
MA_PSA	3.44 ± 0.96	3.42 ± 0.78	3.35 ± 0.94	1.06	0.002	3 < 2 < 1
MA_TA	3.19 ± 1.11	3.13 ± 0.90	3.02 ± 1.08	2.19	0.003	3 < 2 < 1
MA_CA	2.96 ± 1.03	2.73 ± 0.89	2.48 ± 1.07	15.05 ***	0.022	3 < 2 < 1
MAtt	3.02 ± 0.54	3.20 ± 0.52	3.52 ± 0.55	69.04 ***	0.095	1 < 2 < 3
MA_LM	2.85 ± 1.02	3.10 ± 0.88	3.62 ± 0.89	64.07 ***	0.089	1 < 2 < 3
MA_LB	3.34 ± 0.90	3.30 ± 0.74	3.31 ± 0.97	0.11	0.000	2 < 3 < 1
MA_LS	2.86 ± 0.83	3.21 ± 0.67	3.63 ± 0.72	77.14 ***	0.105	1 < 2 < 3

Note: ME means Mathematics engagement; ME_BE means Mathematics engagement_Behavioral Engagement; ME_CE means Mathematics engagement_Cognitive Engagement; ME_EE means Mathematics engagement_Emotional Engagement; MA means Mathematics anxiety; MA_PSA means Mathematics anxiety_Problem Solving Anxiety; MA_TA means Mathematics anxiety_Test Anxiety; MA_CA means Mathematics anxiety_Classroom Anxiety; MAtt means Mathematics attitude; MA_LM means Mathematics attitude_Learning Motivation; MA_LB means Mathematics attitude_Learning Beliefs; MA_LS means Mathematics attitude_Learning Strategies. *** *p* < 0.001.

**Table 6:** The effect sizes of the mediation effects.

	95% Confidence Interval		
	Effect Size (SE)	LLCI	ULCI
Total effects			
M_PTS→Mathematics engagement	0.51 (0.11)	0.29	0.73
H_PTS→Mathematics engagement	1.10 (0.11)	0.88	1.32
Indirect effects			
Indirect effect 1: M_PTS→Mathematics anxiety→Mathematics engagement	0.003 (0.01)	−0.01	0.02
Indirect effect 2: H_PTS→Mathematics anxiety→Mathematics engagement	0.01 (0.01)	−0.01	0.04
Indirect effect 3: M_PTS→Mathematics attitude→Mathematics engagement	0.19 (0.07)	0.06	0.32
Indirect effect 4: H_PT→Mathematics attitude→Mathematics engagement	0.51 (0.08)	0.38	0.65
Direct effects			
M_PTS→Mathematics engagement	0.32 (0.09)	0.14	0.49
H_PTS→Mathematics engagement	0.57 (0.09)	0.40	0.75

Note: Effect Size, standardized effect; SE, standard error; LLCI, lower-level confidence interval; ULCI, upper-level confidence interval.

## Data Availability

The data used to support the findings of this study are available from the corresponding author upon request.
